# Role of Phosphatidyl-Serine in Bone Repair and Its Technological Exploitation

**DOI:** 10.3390/molecules14125367

**Published:** 2009-12-22

**Authors:** Antonio Merolli, Matteo Santin

**Affiliations:** 1 Orthopaedics & Hand Surgery, The Catholic University in Rome, Complesso Columbus, via Moscati 31, I-00168 Rome, Italy; 2 School of Pharmacy and Biomolecular Sciences, University of Brighton, Cockcroft Building, Lewes Road, Brighton BN2 4GJ, UK; E-Mail: m.santin@brighton.ac.uk (M.S.)

**Keywords:** phosphatidylserine, matrix vesicles, bone, biomineralization, artificial implants

## Abstract

In the 1970s, morphological evidence collected by electron microscopy linked mineral deposition (“calcification” or “mineralization”) in newly-forming bone to membrane-encapsulated particles of a diameter of approximately 100 nm (50–200 nm) that were called “matrix vesiscles”. As the characterisation of these vesicles progressed towards their biochemical composition, the role of lipids in the biomineralization process appeared to be crucial. In particular, a group of cell-membrane phospholipids were identified as major players in the crystal formation process. Indeed, in the 1980s it became clear that phosphatidylserine, together with proteins of the annexin family, was among the most important molecules in binding calcium ions and that this phospholipid was involved in the regulation of the early stages of mineralization *in vivo*. During the same period of time, the number of surgical implantations of orthopaedic, dental and maxilo-facial devices requiring full integration with the treated bone prompted the study of new functionalisation molecules able to establish a stable bonding with the mineral phase of the host tissue. In the late 1990s studies started that aimed at exploiting the potential of calcium-binding phospholipids and, in particular, of the phosphatidylserine as functionalisation molecules to improve the osteointegration of artificial implants. Later, papers have been published that show the potential of the phophatidylserine and phosphatidylserine-mimicking coating technology to promote calcification both *in vitro* and *in vivo*. The promising results support the future clinical application of these novel osteointegrative biomaterials.

## 1. Bone structure and function

Bone plays fundamental mechanical and protective functions in vertebrates. These functions are successfully exerted by the unique combination of a mineral phase deposited on a proteic template. Ossification is the formation of bone by the activity of cells termed “osteoblasts” which deposit the proteinaceous matrix (the osteoid tissue) and later promote the deposition of minerals which they take from the blood. This process of mineral deposition is called “mineralization”. Alternatively, the process is also indicated as “calcification” to emphasise the calcium-based composition of the mineral phase [1]. However, osteoblasts are not the sole cell type responsible for the mineralization process: “chondrocytes” (the cells of the cartilage) are able to promote the “endochondral ossification” [2] and “odontoblasts” (the cells of the teeth) produce mineralized dentin [3].

As any other tissue of the vertebrate and human body, bone is a living tissue that undergoes a continuous remodelling process where the older bone is continuously resorbed by the activity of some cells, the so called “osteoclasts”, and gradually replaced by newly-formed tissue deposited by bone producing cells. “Bone turn-over” is the term which defines this continuous cycle of bone resorption and apposition which is driven by these types of cells. Bone turn-over is finely tuned by biochemical signaling (hormonal, autocrine and paracrine) and is the result of a complex series of biochemical and cellular interactions. These events contradict the apparently inert nature of the bony tissue. Indeed, the annual rate of bone turn-over in a healthy individual ranges from 10% to 20% percent of the whole skeleton [1].

Alongside the biochemical signaling, bone is deposited in proportion to the compressive cyclic load that the tissue has to sustain. A microscopic analysis shows that bone deposition follows the lines of mechanical stress. Therefore, each bone segment constantly adapts its inner structure to the applied loads, a process termed “Bone Remodelling” [1,4,5,6]; this phenomenon can be easily seen at work also during the fracture healing process [7,8,9] or during bone regeneration by limb lengthening techniques [10,11]. 

The organic matrix of bone is composed primarily of collagen, a fibre-arranged protein that provides flexibility to the tissue. Collagen accounts for approximately 10% by weight of the adult bone mass. Bone is mainly composed of a mineral phase made of carbonated hydroxyapatite (about 65% of adult bone mass). Water comprises approximately 25% of adult bone mass. Collagen fibers have great tensile strength while the calcium phosphate phase offers compressive strength. These properties are enhanced by the intimate bonding of the organic and mineral phase to provide the unique tensile and compressive properties of the bony tissues [1,12].

## 2. Matrix Vesicles

In 1969, following an electron microscope investigation, Anderson reported the presence of “matrix vesiscles” (MVs) associated with the process of bone calcification [13]. The same author has reviewed this topic after thirty years of research [14,15,16] describing the MVs as extracellular membrane-invested particles of approximately 100 nm (50–200 nm) in diameter. The description highlights that these particles are ‘selectively located at sites of initial calcification in cartilage, bone and predentin’ and that they are the nucleation centres for the early formation of apatite crystals. In particular, it has been reported that these early crystal nuclei are formed within the MVs in close proximity to their bi-layered phospholipid membrane. Anderson proposed that the MVs biogenesis takes place at ‘polarized budding and pinching-off of vesicles from specific regions of the outer plasma membranes of differentiating growth plate chondrocytes, osteoblasts, and odontoblasts’. More recently, he reported how MVs initiate and promote also a variety of forms of pathological calcification [16]. Polarized release of MVs into selected areas of the developing bone matrix prevents the non-random distribution of calcification. The early formation of crystal nuclei within the MVs (phase 1) is enhanced by the activity of both specific enzymes phosphatases, *i.e.,* alkaline phosphatase, adenosine triphosphatase and pyrophosphatase, and by calcium-binding molecules like those of the Annexin family (AnxA1, AnxA2, AnxA5 and AnxA6) and like phosphatidylserine (PS); all these macromolecules are localised within or near to the MV membrane. The so-called Phase 2 of the biomineralization begins with the release of the crystals from the MVs, a process that exposes the preformed hydroxyapatite crystals to the extracellular fluid. The extracellular fluid normally contains sufficient Ca^2+^ and PO_4_^3-^ to support continuous crystal growth, with the preformed crystals serving as nuclei (templates) for the formation of new crystals by a process defined as homologous mineralization.

## 3. Phospholipids and Calcification

**Figure 1:**
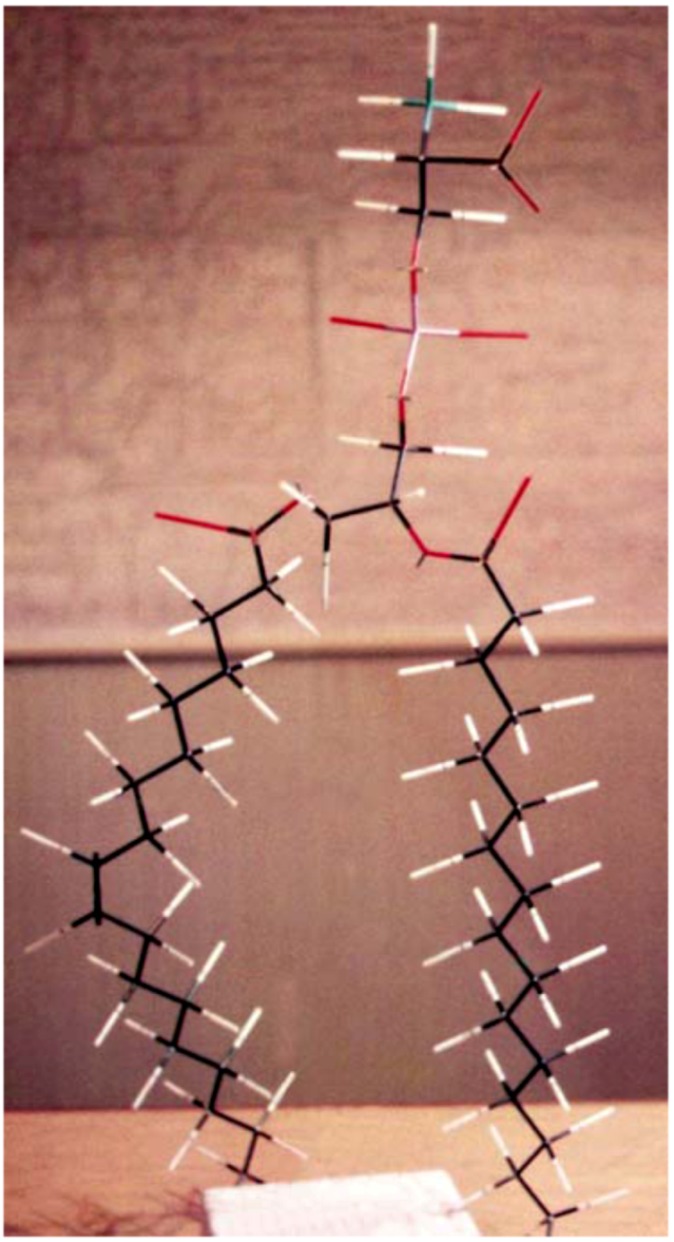
A “skeleton” model of 1-palmitoyl-2-oleoyl-SN-glycero-3-phospho-L-serine built by the first author in 1981. The carbon tail on the right is shown partially.

In the first part of the 1970s a series of papers were published that focused on the characterisation of the MVs and on their role in initial stages of calcification [17,18,19,20,21,22,23,24,25]. At the same time, other investigators evidenced the role of lipids in calcification [26,27,28,29,30,31]. In 1976 Vogel wrote that two current areas of research on hard tissues were focused upon: (a) acidic lipids associated with the local mechanism of calcification, and: (b) the presence of MVs as the loci for initial mineralization. The author concluded that “data which show that acidic phospholipids are a component of the vesicle membrane provide a common denominator between these two areas” [32]. Studies, then, converged on this “lipid-induced calcification” [33] and several phospholipids, in particular PS, were found to play a key role (Figure 1).

It was highlighted that MVs had an overall lipid content higher than either the membrane or cell fractions and that they were significantly enriched in serine-phosphoglycerides and cholesterol. Despite their different composition, these data supported the view that they were derived from the plasma membrane of the cell as earlier conclusions based upon morphological and enzymological evidences had already suggested [28].

To study the effect of phospholipids on the conversion of amorphous calcium phosphate (ACP) to crystalline hydroxyapatite (HA), *in vitro* experiments were conducted in the presence or absence of PS and other phospholipids. In particular, it was demonstrated that PS and other phospholipids possessing either anionic or negatively-charged zwitterionic properties were able to bind calcium and that they were effective in stabilising ACP. These data were extrapolated to suggest that lipids may play a role in the control of physiological mineralization *in vivo* [29].

Histochemistry studies showed that areas of early mineralisation were intensely stained by lipid-specific dyes. Later, these lipids were isolated and biochemically characterised as phospholipids. The two predominant species were PS and phosphatidyl inositol (PI). Therefore, it was proposed that these phospholipids were among the most active nucleation sites of apatite crystal formation in MVs [30]. In 1976 Wuthier reported that MVs were rich in cholesterol, free fatty acids, sphingomyelin, glycolipids, lysophospholipids, and PS, while they were depleted of phosphatidylcholine and ethanolamine. As indicated by Anderson, Wuthier also reported that that MVs originate from a budding process of the chondrocyte surface membrane. Furthermore, Wuthier highlighted that metabolic studies had indicated that the MVs formation is a relatively rapid biochemical process (*i.e.,* less than 6 hours) involving both lipid synthesis and degradation [31]. Wu, in 2002, reported on MVs composition showing that phosphatidylserine forms complexes that accompany mineral formation, while degradation of other membrane phospholipids apparently enables egress of crystalline mineral from the vesicle lumen [34]. 

## 4. A Role for Phosphatidyl-Serine

Although electron microscopic morphological studies have continued until recently [35,36,37], the bulk of research has focused on a full characterisation of the structures responsible for the early stages of calcification. These studies seemed to indicate that this process occurs within the MVs because of a more complex assembly of proteins and lipids; some authors have termed these assemblies as “nucleators”. The nucleator of bone matrix calcification was described as a protein-phospholipid complex where phospholipids were identified as mono and diphosphoinositides and PS [38]. Between these two macromolecules, PS was identified as the major phospholipid constituent [39]. Parathyroid hormone was found to affect the calcium/PS stimulated phosphorylation of proteins in bone [40] and the enrichment of MVs by PS was correlated with the rapid accumulation of Ca^2+^ in the MV interior [41].

Subsequent work gradually led to the identification of the proteins of the lipid-protein complex. A quantitatively prevalent group of the MV proteins, the acidic phospholipid-dependent Ca^2+^-binding proteins (APD-CaBP), were found to be immunologically related to the annexin family [42].

Calcifiable proteolipids were postulated to enhance apatite deposition by structuring membrane PS molecules into a conformation conducive to mineral formation. Annexin and anchorin CII, a protein known to bind type II collagen, were closely correlated [43]. A further characterisation highlighted the presence of annexin V, a type of ion-selective Ca^2+^ channel protein that possesses selective collagen binding properties [44] and can be defined as a PS-dependent Ca^2+^-binding protein; it was found to be the major protein in the nucleation core [45]. Other investigations have established that calcium uptake by MVs depends on the binding of annexin V to collagen type II and type X and that annexin V plays a major role in the MV-initiated cartilage calcification as a collagen-regulated calcium channel [46].

In 1997, while studying the process of calcification in liposomes (artificial membranous lipid vesicles), Kirsch speculated that since the lipid composition of matrix vesicles is rich in PS, it may regulate annexin V function. In particular, a significant Ca^2+^ influx in presence of annexin V occurred only in liposomes containing a high PS content. His studies indicate that PS-rich bilayers induce the formation of a hexameric annexin V, possibly leading to a Ca^2+^-dependent insertion of annexin V into the phospholipid bilayer and to the establishment of annexin V-mediated Ca^2+^ influx into MVs or liposomes [47].

To emphasise the important role played by MVs in biomineralization, Genge, Wu and Wuthier have recently stated that MVs “are involved in *de novo* mineral formation by nearly all vertebrate tissues”. These authors have summarised the biomineralization potential of these structures in the composition of their nucleation core that they reported to be composed of three principal interacting constituents: (i) the amorphous calcium phosphate (ACP), (ii) calcium-phosphate-lipid complexes (CPLX) formed by ACP partially combined with PS and (iii) annexin A5 (AnxA5). AnxA5 synergistically activates PS-CPLX transforming it from a very weak nucleator of mineral formation into a potent nucleation site [48,49,50,51].

Other proteins than the annexin family can bind to PS such as the milk fat globule epidermal growth factor 8 (MFG-E8) [52,53,54,55]; a product of the growth arrest-specific gene 6 (Gas6) [55,56,57,58]; the developmental endothelial locus-1 (Del-1) [59]; the serum-derived protein S [60,61]; the T cell immunoglobulin mucin protein 4 (Tim-4) [62,63,64,65,66]. Although, there are no experimental evidence of possible implications of these proteins in matrix-vesicles-induced mineralization, one can not exclude that they may be implicated in cell-induced and/or extracellular-induced mineralization.

## 5. Technological Application of Artificial MVs

It is widely accepted that the integration of artificial implants into the treated bone depends on the ability of the material to support the early apposition of newly synthesized bone at the implant surface. In 1995, Braun placed titanium implants in the right tibias of rats following ablation of the marrow showing that MVs and PS content as well as alkaline phosphatase and phospholipase A2 activity were increased showing a peak after 6 days. However, the same study highlighted that these levels were lower than those observed in bone healing in absence of the implant [67].

In 1998, Taylor synthesized a PS-amorphous calcium phosphate complex as a model of the matrix vesicle system [68]. In 2000, Sela studied osteogenesis around implants and observed that this process depended on the migration of osteoprogenitor cells to the implant site as well as to their ability to synthesise and secrete a mineralising extracellular matrix. The study concluded that as osteogenesis around implants is similar to the new bone formation, the mechanism by which the cells calcify their matrix involves MVs in a process of "primary mineralization" [69]. Parallel studies proposed liposomes as model structures for biological calcification processes and showed that these structures were able to promote calcification and accelerate the formation of bone-like tissue [70].

In 2002, Camolezi and co-workers standardized a method for an alkaline phosphatase-liposome system to mimic matrix vesicles. The 395 nm diameter of the alkaline phosphatase-liposome system was relatively homogeneous and stable when stored at 4 degrees Celsius and by this system the kinetic behavior of the incorporated enzyme was examined *in vitro* [71].

### 5.1. Calcium-binding phospholipids as coating molecules of medical implants: Early evidences of their osteointegrative potential

*In vitro* coating of implantable materials was experienced to evaluate the effect of different phospholipid coatings on osteoblast responses. In 1998, the team at the School of Pharmacy and Biomolecular Sciences, University of Brighton, UK, started a collaborative project (acronym: Lipostin) sponsored by the European Commission that involved partners from different European countries. This project soon led to the developing and intellectual property protection of a novel method of coating of implants with calcium-binding phospholipids such as PS and PI (A.W. Lloyd, M. Santin, W.G. Love, S.P. Denyer, W. Rhys-Williams. Biomedical Implants, 2000, PCT/GB00/03290 [72]). Different formulations were optimised that were able to form a 3D hydrogel interpenetrating the porous surface of medical-grade titanium implants and to induce a rapid mineralization of their surface upon incubation in simulated body fluids (SBFs).

Later, by a similar approach, Satsangi tested commercially available phospholipids (phosphatidylcholine, PS and PI) converting them to their Ca-PL-PO(4) and coating commercially pure titanium grade 2 disks [73]. Using uncoated Ti surfaces as controls, cell responses to phospholipid-coated surfaces were evaluated using the American Type Culture Collection CRL-1486 human embryonic palatal mesenchymal cells, an osteoblast precursor cell line, over a 14-day period. Titanium surfaces coated with PS exhibited enhanced protein synthesis and alkaline phosphatase specific activity compared to other phospholipids and uncoated surfaces [73]. The, polar head group of phospholipids in coated surfaces was observed to have an influence on the calcium deposition as well as the osteoblast differentiation. Among the phospholipids evaluated, PS exhibited the strongest calcium deposition and more enhanced alkaline phosphatase specific activity [74].

Susin and co-workers recently followed a similar approach with phosphorylcholine (PC); they coated titanium implants and analyzed *in vivo* in the rabbit the biomechanical (removal torque) and histomorphometrical results. They did not find any significant differences in bone density with control implants, nevertheless they concluded that PC coating technology appears to be a viable candidate delivery system for agents in support of local bone formation at endosseous implant surfaces [75].

Following protection of the intellectual properties, a series of papers were published by the team at the University of Brighton that showed the full potential of this technology both *in vitro* and *in vivo*. Santin *et al*. showed that in PS-based coatings, deposited on titanium coupons by dip-coating, upon dehydration in a simulated body fluid lead phospholipids to quickly crosslink by calcium ions and re-arranged into a three-dimensional matrix able to induce rapid formation of a calcium phosphate mineral phase. In the attempt to closely mimic the cell membrane composition, heterogeneous formulations based on the mixing of anionic phospholipids (either PS or PI) with phosphatidylcholine and cholesterol were synthesized. However, homogeneous PS coating was a more effective calcification environment than the heterogeneous formulations [72]. Other studies suggested that in addition to improving the nucleation process for new bone formation, coating titanium with phospholipids may reduce the inflammatory response, which was shown to vary depending on the formulation employed. When compared to uncoated titanium and HA-coated implant, PS significantly reduced the adhesion of the inflammatory cells, *i.e.,* the monocytes/macrophages [76]. It has been speculated that this lack of adhesion is caused by the natural role played by PS during the phagocytosis of apoptotic cells. Indeed, during the programmed cell death, the apoptosis, the PS molecules of the plasmalemma bilayer flip from the cytoplasm side of the membrane towards the cell surface. This presentation of PS molecules to the cell surface is recognized by the macrophages as a signal for the clearance of the dead cells by phagocytosis. In the case of PS-coated implants, the relatively large size of the surface does not allow the macrophages to phagocyse the foreign body; this fact produces an actual inhibition of macrofages adhesion and activation, thus provoking an eventual anti-inflamatory action of PS coating. The biocompatibility of these phospholipid-based coatings, in combination with their ability to initiate rapid mineralisation, showed that these coating could promote bone cell interactions *in vitro* and, as a consequence, had a promising potential to support implant osteointegration *in vivo* [77].

### 5.2. In vivo response to phosphatidyl-serine coating

The promising features showed by the *in vitro* experiments on titanium implants coated with calcium-binding phospholipids prompted the validation of their osteointegrative potential *in vivo*. The aim of the *in vivo* study was to assess the ability of these biomaterials to offer an alternative to traditional osteointegrative coatings such as those based on bioactive ceramics. Indeed, bioactive ceramics such as HA are employed as coatings to enhance the fixation of metallic devices; they act as a scaffold to enhance bone formation on their surface. The osteointegrative potential of synthetic HA (the most widely used bioactive ceramic material in surgery) is linked to its chemical nature which is very similar to that of the bone mineral phase thus providing an optimal substrate for the growth of the osteoblasts [78]. The discovery that osteoblasts can grow easily on artificial HA, both as a bulk material and as a plasma-spray coating, improved significantly the possibility of obtaining a favorable response to the implantation of devices that were loaded or coated with HA. Not only are osteoblasts found growing on HA, but osteoclasts also are able to resorb it thus involving the biomaterial in the physiological bone turnover [79]. For these reasons, HA has rightly been considered as a biomimetic biomaterial [80] and coating an implant with HA has become an effective system to mask the foreign body nature of the underlying implant material. Nevertheless, drawbacks are associated with the use of HA. In particular, highly crystalline HA (crystallinity of 85%, according to the method of Tudor *et al*. [81]) renders the coating brittle under biomechanical stresses and prone to a slow degradation into particles [79], while lower crystalline coatings is prone to a relatively rapid degradation in body fluids [82].

Therefore, PS-based coatings seemed to offer an opportunity to generate a rapidly mineralizing substrate at the bone/implant interface. To prove this, an *in vivo* study using a well-established rabbit femoral model was adopted. The osteointegration of porous titanium implants coated with either PS or phosphatidyl choline/PS/chloresterol formulation was compared by the use of high-magnification back-scattering scanning electron microscopy (BSEM) to uncoated titanium as well as to conventional plasma spray HA coatings. Results showed that PS-based coatings were gradually resorbed while being able to promote the apposition of new bone to the metal surface at level higher than bare titanium [83].

**Figure 2:**
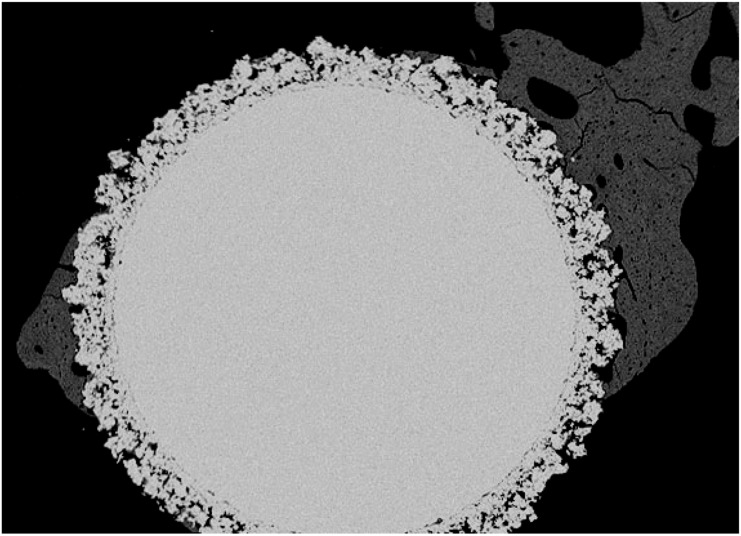
BSEM transverse section of a PS-coated titanium foam cylinder after eight weeks of implantation into a rabbit femur (magnification 15×).

In particular, after four weeks implantation, the control Ti shows bone trabeculae growing towards and around the implant. High magnification of the bone/implant interface, however, showed that the new bone did not establish a direct contact with the material surface. At eight and 26 weeks the titanium foam allowed the penetration of a healthy and remodelling trabecular bone throughout its porosity, but still showed areas of incomplete apposition of tissue in several specimens when analysed at high magnification. In the case of the PS-coated implants, the low magnification analysis after four weeks of implantation shows a bone formation with trabeculae growing towards the surface of the implant. The newly-formed bone was not adjacent to the implant surface when analysed at high magnification. After eight weeks, however, the newly-formed trabecular bone in-growth had invaded almost completely the porous implant establishing a direct contact with its surface in most of the areas analysed (Figure 2).

**Figure 3:**
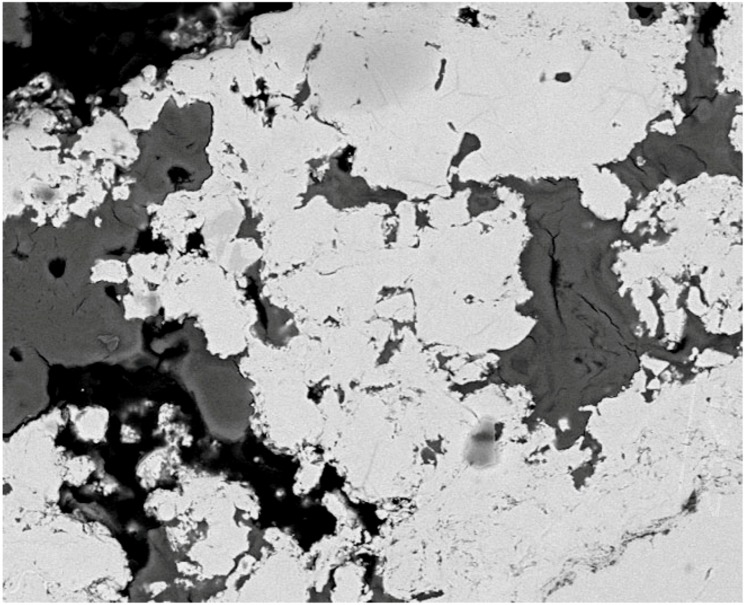
BSEM transverse section of a PS-coated titanium foam cylinder after 26 weeks of implantation into a rabbit femur (magnification 250×).

Tissue morphology at the implant surface was observed to be normal after 26 weeks when the invasion of the tissue was still readily visible and its apposition to the implant surface consolidated (Figure 3).

The relatively slow degradation of the phospholipid coatings was obviously not able to match the fast rate of apposition of bone to the HA surface. However, taken into account the clinical problem associated to the delamination of HA coatings from the surface of metal implants, the ability of the phospholipid coatings to mediate a final tight apposition of bone to the metal implant is likely to offer a long-term clinical benefit. These promising results can be explained by the data collected *in vitro* showing the fast mineralization of the phospholipid coatings and their good substrate properties for osteoblast adhesion [72,76,77]. It can be speculated that the mineralization potential and resorption of calcium binding-phospholipids can gradually drive the in-growth of new bone tissue towards the implant surface allowing a final, intimate contact between the mineral phase and the metal. Furthermore, the lack of adverse fibrous reaction confirmed the ability of the phospholipid coatings to control the inflammatory response to the implant.

## 7. Conclusions

During a period of about thirty years PS has been recognized as one important molecule involved in the mineralization process in bone formation. When exploited as a possible functionalisation molecule for artificial implants, this new type of biomimetic (matrix vesicle-like) biomaterial was able to promote the osteointegration of titanium implants. PS coating, uniquely re-arranged into 3-D porous gels when immersed in buffer simulating the body fluid composition showing their potential to prevent the formation of non-mineralised tissue *in vivo*. These phospholipid-based coatings have also shown the ability of affect other important component of the bone repair process, the osteoblast and the inflammatory cells. Osteoblast adhesion and proliferation was clearly enhanced on the surface of titanium implant coated with PS, while inflammatory cell adhesion was inhibited. Therefore, PS coatings seem to fulfil all the main characteristics required to the functionalisation of medical implants where osteointegration is required. However, this technology based on calcium-binding phospholipid can also be exploited to produce tissue engineering scaffolds. Recently a paper has been published about a composite scaffold for potential use in bone tissue engineering, to examine the *in vitro* reponse elicited in human mesenchymal stem cells (hMSCs); this novel biomimetic scaffold is based on a mix of bioactive glass–collagen–hyaluronic acid-PS [84].

These encouraging research projects prompt the use of this technology in clinical applications where bone repair needs to be pursued by the means of highly performing, safe and commercially sustainable biomaterials.
